# Neural Mechanisms and Information Processing in Recognition Systems

**DOI:** 10.3390/insects5040722

**Published:** 2014-10-13

**Authors:** Mamiko Ozaki, Abraham Hefetz

**Affiliations:** 1 Department of Biology, Graduate School of Science, Kobe University, 1-1 Rokkodaicho, Nada, Kobe 657-8501, Japan; 2 Department of Zoology, George S Wise Faculty of Life Sciences, Tel Aviv University, Ramat Aviv 69978, Israel; E-Mail: hefetz@post.tau.ac.il

**Keywords:** nestmate recognition, neural template, sensory adaptation, ants, chemosensillum, antennal lobes, mushroom bodies

## Abstract

Nestmate recognition is a hallmark of social insects. It is based on the match/mismatch of an identity signal carried by members of the society with that of the perceiving individual. While the behavioral response, amicable or aggressive, is very clear, the neural systems underlying recognition are not fully understood. Here we contrast two alternative hypotheses for the neural mechanisms that are responsible for the perception and information processing in recognition. We focus on recognition via chemical signals, as the common modality in social insects. The first, classical, hypothesis states that upon perception of recognition cues by the sensory system the information is passed as is to the antennal lobes and to higher brain centers where the information is deciphered and compared to a neural template. Match or mismatch information is then transferred to some behavior-generating centers where the appropriate response is elicited. An alternative hypothesis, that of “pre-filter mechanism”, posits that the decision as to whether to pass on the information to the central nervous system takes place in the peripheral sensory system. We suggest that, through sensory adaptation, only alien signals are passed on to the brain, specifically to an “aggressive-behavior-switching center”, where the response is generated if the signal is above a certain threshold.

## 1. Introduction

The chemical language of social insects constitutes a major means of communication that encompasses every aspect of social life [1,2,3,4]. Its complexity varies from simple messages such as alarm pheromones that elicit a single-faceted behavior of rushing with aggression to the emission point, via more complex messages such as trail pheromones that encompass several levels of information and evoke complex as well as context-dependent responses, and up to the very complex pheromones responsible for nestmate recognition (Here we use the term pheromone in the broader sense as first defined by [5], but see [6] for the term “signature mixtures” with respect to nestmate recognition). These levels of informational complexities are reflected both in the chemical complexity of the message and presumably in the complexity of the neural circuits that decipher the information. We therefore briefly discuss several aspects of the chemistry of semiochemicals and the biological meaning of blend complexity before addressing the neural deciphering mechanisms.

Alarm pheromones need not be highly specific either at colony or even at species levels, and, therefore, they can theoretically be composed of only a few compounds or even a single one. In cases in which the alarm pheromone comprises several compounds the response to each of them seems to be independent, and the sensing of an individual component evokes only the respective behavioral component [7]. The lack of necessity for specificity has undoubtedly affected the evolution of the signal, in terms of both its biosynthesis and its perception. For example, adoption of a metabolic end product as an alarm pheromone is subjected only to certain physical constraints: namely, it has to be volatile, enabling the message to disseminate rapidly into the immediate environment and to disappear rapidly if not reinforced. Perception of and reaction to the pheromone has a clear advantage to conspecific ants, irrespective of colony origin and therefore can readily be fixed in the population. Perception of alarm pheromones may also confer advantage upon other ant species in the population that might have similar enemies, which explains the lack of species-specificity often found with these signal types.

At the other extreme are the pheromones responsible for recognition of nestmates, resulting in discrimination between friends and foes. Here specificity is highly important and the information conveyed needs to be very accurate. This poses a great evolutionary challenge because the pheromones must be very complex in order to be colony-specific [8]. The major problem is that, unlike alarm pheromones where trait uniformity is advantageous because all individuals in the population can use and benefit from such uniformity, within-population trait-diversity must be the rule in a nestmate recognition system.

## 2. A Synopsis of Nestmate Recognition in Ants

### 2.1. The Signal

The biology and mechanisms of nestmate recognition have been extensively reviewed in the past 15 years [9,10,11,12]. Below is a brief account of the processes relevant to the complexity of the signal and, accordingly, the required complexity of the neural system for deciphering the message.

There are several features that have shaped the properties that characterize nestmate recognition through chemical signals. Generally, the recognition pheromones are perceived upon physical antennal contact with any part of the body surface of the encountered ant [10], or at least are perceived (and recognized) at very close proximity to the encountered individual [13]. Thus, the recognition pheromones are non-volatile, or possess very low volatility, and are widespread over the ant’s body surface. The likely candidates are the cuticular hydrocarbons (CHCs), which are ubiquitous among insects and present highly complex mixtures in ants. The original function of the CHCs is assumed to be in creating an impermeable layer that protects the insect from desiccation [14,15], the efficacy of which depends on their molecular weight and degree of branching [16]. Theoretically, selection for an effective impermeable layer should result in a linear hydrocarbon with carbon length of over 25. However, in most insects, and particularly in ants, the occurrences of hydrocarbon mixtures, of unsaturated hydrocarbons and of branched hydrocarbons, all reduce the efficacy of this layer as an impermeable barrier. This suggests a trade-off between the cost of having a less efficient impermeable barrier and the benefit of adopting the externalized hydrocarbons for other functions, e.g., communicative function [10]. A study on *Pogonomyrmex barbatus*, for example, revealed that forager workers have augmented amounts of linear hydrocarbons compared to in-nest ants, in line with the former’s needs for greater protection from desiccation [17], and emphasizing the above postulated trade-off.

As noted above, ants possess highly complex mixtures of CHC, generally ranging from 21-carbon chain-length to above 40. In all the species studied so far, the CHCs may communicate both colonial identity (nestmate recognition) and reproductive caste (fertility signaling) [10,11,18,19]. This dual function may create a possible conflict, because their role in nestmate recognition necessitates colonial uniformity, whereas caste specificity implies different compositions for different members of the colony. There are several ways by which these two contrasting requirements can reconcile. One is through the use of different subsets of hydrocarbons, or even a single component from the blend, for delineating colonial identity and caste/task function [20,21,22,23]. In terms of perception this necessitates two disparate systems that act independently, or two different neural networks in the brain, yet to be demonstrated. An alternative could be a threshold- based response that is also context dependent [10,18]. Large differences in composition indicate an encounter with a heterospecific or heterocolonial individual; while smaller differences may indicate caste specificity and so forth. This hypothesis neither necessitates a disparate perception system nor a different neural network, merely a threshold-dependent response.

Direct evidence for the selective role of CHCs in nestmate recognition has been shown in many ant species [24,25,26,27,28,29]. The behavioral tests designed to quantify the responses between nestmate *vs.* non-nestmate comprise situations in which a nestmate anointed with a non-nestmate odor is abnormally aggressed, while the usually high aggression towards a non-nestmate is reduced if the latter is anointed with a nestmate odor. However, it is important to note that the response towards a nestmate anointed with a non-nestmate odor is much more pronounced than the decline in aggression when anointing a non-nestmate with a nestmate odor. This indicates that the ants are much more sensitive to differences than to similarities in the nestmate recognition pheromone composition. This finding has major implications for our understanding of the perception and deciphering systems, as we describe in detail below.

### 2.2. The Template

Signal deciphering requires not only its perception but also an internal representation to which it is compared. This is the template [30]. The template may be genetically determined and heritable: for example, the specific alarm calls of prairie dogs when detecting either a terrestrial or an aerial predator [31], and the deterrent effect of a coral snake image on naïve hand-reared motmots [32]. In these cases the template can be a fixed attribute and therefore reflect simple heritability. When the information is more complex, for example in mate choice, another layer must be added to the template. The first layer is the recognition of male ornaments and/or other male-specific characteristics, which requires an innate, thus heritable, template. However, for assessing male quality a quantitative and/or qualitative component must be added, whose assessment should rely on a comparison between males and, therefore, involve learning and consequent template adjustment. For example, in mice the recognition of male urine may involve a fixed heritable template, whereas recognizing whether it is a highly competitive male [33,34], or assessing its health status [35], requires a more plastic template. When more complex information must be conveyed, for example recognition of kin, social partners, or individual status, even more specific information is required and the template is thought to be acquired by learning at an appropriate stage in the individual’s ontogeny [36,37].

Social recognition is imperative for maintaining cooperation and social cohesion, exerting high selection pressure on the accuracy of recognition and consequently on the acuteness of the template. The acuteness of the template is a balance between the cost and benefit of error in recognition [36,38]. In terms of sociality, if the cost of rejecting a desirable individual, e.g., a nestmate in social insects, exceeds that of accepting an undesirable individual, e.g., a selfish exploiter individual or a social parasite in social insects, then small discordances between the signal and template are acceptable. If, on the other hand, the cost of accepting an undesired individual is greater than rejecting a desirable individual, even small mismatches between signal and template are unacceptable.

Early theoretical consideration of the factors shaping recognition systems engaged with kin recognition and centered around genetic models and recognition alleles [39,40]. The general idea was that two individuals bearing the same recognition alleles could identify one another through phenotype matching. However, it is questionable whether social insects actually use kin recognition in shaping their behavior. The hypothesis that sociality in insects evolved through kin selection [41,42] predicts the concomitant evolution of kin recognition [43]. Since the ancestral social insects were typically monogyne and monandrous [44] all members of the colony were similarly genetically related, so that kin recognition equaled nestmate recognition. However, as variations in both the number of queens and the switch from monoandry to polyandry evolved, kin selection may have been counteracted by colony level selection and, consequently, nestmate recognition replaced kin recognition. Most of the studies involving recognition in ants to date test nestmate recognition rather than kin recognition [9]. To account for the genetic variability within the insect society it was postulated that the colony odor represents a mixture of the recognition alleles that its members carry, the genetic *gestalt*, and that the resulting recognition pheromones are transferred among all members of the colony [40], the chemical *gestalt*. Subsequent studies have corroborated this theoretical hypothesis by explicit experimentation [45]. The above-described evolution of the recognition signals was undoubtedly accompanied by an evolution of the perception and deciphering systems, *i.e.*, the template. Rather than being an innate, genetically-based template to match the recognition alleles, it has become acquired.

Several lines of behavioral evidence support the hypothesis that the template in ants is acquired (but see alternative hypothesis below). Newly emerged individuals that are experimentally transferred from their own nest to another nest are readily accepted as nestmates. Moreover, callow workers of different species can be reared together to create a mixed-species colony [46,47,48]. Such mixing is possible because upon emergence the workers lack recognition cues on their body surfaces, which acts as a “clean slate” [9]. By a few days post-emergence, however, the ants have acquired the typical nest signature rendering such transfer impossible [49]. Supporting the clean slate hypothesis is the finding that the ants in a mixed-species group possess a combined CHC composition of both species [50]. Concomitantly with the colony odor acquisition there is template formation in the brain. Thus, the template formation is likely to become established during a critical period after emergence. Support for this type of template acquisition comes from the study of a mixed-species group, where the ants became familiar with the heterospecific odor and aggression towards the heterospecifics reflected gradation according to the degree of similarity [48,51]. However, unlike imprinting that is generally fixed, the template for nestmate recognition in ants does not remain fixed following initial acquisition, but is updated to match dynamic changes in the colony-specific CHC pattern [52,53]. Such continuous template reformation is analogous to the rewriting of stored learned memory [54]. In line with template acquisition through standard associative learning experiment is the report that workers of *Pristomyrmex punctatus* learn to associate CHCs of mutualistic *Narathura japonica* caterpillars with food rewards and, as a result, are more likely to tend to these caterpillars [55]. However, the workers do not perform such an associative learning of the CHCs of a non-ant-associated caterpillar such as the lycaenid, *Lycaena phlaeas*, even if rewarded artificially, which suggests that although ants have the ability for associative learning of CHC blends, it is still constrained by evolutionary factors. That response is contextual even under associative learning was demonstrated in the ant *Camponotus aethiops* that although learned to associate complex blend of hydrocarbons with food were still aggressive to ants bearing such a blend [56]. We suggest that similar associative learning of nestmate CHC by callow workers while being groomed or fed by trophallaxis may be involved in the formation of the primary template for nestmate recognition. Noteworthy, since callow workers do not have yet the full complement of CHC [57], the prefilter system is non-operative yet and therefore does not interfere with the primary template formation. Trophallaxis among mature nestmate workers following mutual antennation may also largely contribute to associative learning of the nestmates’ CHC odor, thereby both updating the template and consolidating it.

## 3. The Neuroanatomy of the Signal Perception and Deciphering Systems

As described above, nestmate recognition in ants is generally attained following physical antennal contact with the body surface of the encountering ant [10], although it was shown that in *Camponotus floridanus* perception of a non-nesmate hydrocarbon-blend can be attained also from a distance of about 1 cm without actual antennal contact with the ant surface [13]. This suggests that perception might involve contact chemosensilla, similar to the many gustatory sensilla common on the antennae of many insects, ants included. Such sensilla that are specifically sensitive to CHCs were described in *Camponotus japonicus* [27], but unlike the many gustatory sensilla that are characterized by a single top pore, the CHC sensilla have numerous tiny pores, which are characteristic of insect olfactory sensilla. These CHC sensilla are 4 μm in diameter and 20 μm length and are morphologically categorized as sensilla basiconica [58], and are abundantly distributed in the distal segments of the antennae in workers and queens but not in males [59]. The cuticular shafts of the CHC sensilla are perpendicularly oriented to the antennal surface, which facilitates chemical inspection of encountered objects. Stimulant molecules penetrate the cavity of the sensillum through these tiny pores, where they are trapped by olfactory receptor neuron (ORN)-specific receptor molecules located on the membrane of the dendritic processes of the ORNs.

In ants, as in many other insects, the highly hydrophobic pheromones are carried through the hydrophilic milieu bathing the ORNs through the mediation of lipophilic ligand-binding proteins. These include odorant-binding proteins (OBPs) [60,61] chemosensory proteins (CSPs) [62,63] and the recently reported Niemann–Pick type C2 protein (NPC2) [64]. The latter were found to accumulate in the CHC sensilla basiconica of the ant *C. japonicus*. The main CSP of *C. japonicus* workers, CjapCSP (CSP7 in [63]), dissolved *in vitro* the CHC components in buffer solution at the same ratio as the original CHC blends, indicating that also *in vivo* the protein conserves the colony-specific CHC composition that reaches the ORN [27]. Due to such transport fidelity, the constant transport of self-CHCs into the ORNs will specifically desensitize the respective ORN, whereas non-nestmate CHCs that sporadically reach the sensillar milieu will elicit a neural response in the ORN. The evidences that are either supportive or negative of the idea of sensillar desensitization with respect to nestmate recognition are discussed in the section that explains the prefilter hypothesis.

Ants constantly clean their antennae with a special antennal cleaner located in their front legs [65,66]. Cleaning is often accompanied by passing the front legs through the mouthparts, presumably to absorb the antennal CHC accumulated in the cleaner, but also possibly applying new CHC from the postpharyngeal gland (PPG). The importance of the antennal cleaning became evident in a recent study of the cockroach *Periplaneta americana*, revealing that the CHCs secreted onto the antennae are appropriately cleaned by self-grooming to maintain correct responsiveness of the olfactory sensilla [57]. Workers of *C. japonicus*, if prevented from self-grooming of the antennae, might not exhibit aggressive behavior, either towards nestmates or non-nestmate workers. It is suggested the need for the maintenance of the cuticular surface or outer microenvironment of the CHC sensillum by self-grooming in order to maintain proper functioning of the sensory prefilter system.

Upon perception, the generated neural signal reaches the brain via axonal projection from the ORN to the multiple glomeruli of the AL. The neural network is constructed in such a way that functionally identical ORNs, irrespective of their topology in the antenna, converge to the same glomerulus [67,68,69]. This enhances sensitivity while retaining the idiosyncrasy of the information. The total number of glomeruli in ant AL, e.g., about 460 in *Camponotus floridanus* [67] and 480 in *C. japonicus* [59], is much larger than in *Drosophila melanogaster* (43 glomeruli/AL) [70] and the honeybee *Apis mellifera* (170 glomeruli/AL) [71]. In workers of *C. floridanus*, the axonal terminals of the antennal ORNs group into seven glomerulus regions, T1-T7 [67]. It was further shown *in* *C. japonicus* that the ORNs derived from sensilla basiconica (the CHC sensilla) converge to the T6 region, which houses more than 30% of the total number of glomeruli in the AL [59]. In the glomeruli the olfactory information can be further processed by the local interneurons, after which it is transferred via projection neurons to a higher neural system, the mushroom bodies (MB) and the lateral horn (LH) [69]. Detailed studies, using fluorescent tracing, of the neuronal tracts from the AL to the higher brain in *C. floridanus* demonstrated that the projection neurons (PNs) with axons leaving the seven distinct glomerulus regions, T1-T7, target different areas in the MB calyx via medial (m-ACT) and/or lateral- antennocerebral tract (l-ACT); In *C. floridanus*, T1-T4 glomeruli are connected to two parts of the MB calix, called lp-II and lp-III; while T3 and T5-7 glomeruli are connected to both lp-I and lpII [67,68]. In contrast, a study in *C. japonicus* revealed that colony odor input from the basiconic sensilla converges into the T6 glomeruli [69], which, in turn, are connected to lp-I, while the non-T6 glomeruli are connected to lp-II. Thus, the information that converges into the T6 glomeruli is sent on to the MB-calyx and a special part of the LH (LH-II) through the m-ACT. This circuiting distinguishes the information regarding colonial identity from other olfactory information, which is mediated via glomerular regions other than T6 (non-T6 region).

## 4. The Neural Template Hypothesis: Putative Neural Mechanisms Underlying Nestmate Recognition

The neural template hypothesis posits that upon perception of the CHC, whether that of nestmate or non-nestmate, the perceiving sensilla send output nerve impulses to the brain where they form a temporary neural network that is compared to a preexisting neural network that constitutes the template.

The deciphering of recognition signals according to the neural template hypothesis comprises four steps of neuronal events: the first two steps involve the acquisition, via the sensory system, of qualitative and quantitative information regarding own colony CHC profile, and memory formation and storage of the *gestalt* colony CHC profile in a brain center, which constitutes the template. Neither colony odor nor the template are hard wired but may change with time. The subsequent two steps involve the acquisition of analytical information of the CHC profile of an encountered ant and its comparison with the preformed template at the higher brain-center, *i.e.*, lateral horn or mushroom body. However, we cannot exclude the antennal lobes as possible decision-making site, in particular when rapid decision has to be taken, such as nestmate recognition [72]. In mice, females reject unfamiliar males based on memorization of the stud male pheromone, which takes place in a particular neuronal circuit of the accessory olfactory bulb, a primary olfactory center [73]. Hence, it is possible that also in ants the antennal lobes might also be responsible for learning and memory concerning with nestmate recognition as well as phenomena like the dear enemy effect. Since the CHC profile differences among conspecific workers are only quantitative (the proportion of each component within the blend), there must be a mechanism that translates these quantitative variations into specific neural circuits. We have little knowledge regarding either how the precise ratio of each CHC component in the blend is preserved in the nerve impulses outputted from the sensilla, or the means by which template comparison is attained.

As described above, a newly-eclosed adult ant presents a “clean slate”. It has only minute, close to zero, amounts of CHC [9,57], and presumably a very flexible template. The ability to compose mixed-species groups of ants reinforces the clean slate hypothesis [46]. However, the fact that phylogenetically distant species often fail to compose a mixed-species group, also indicate a possible innate, genetically predetermined, propensity for recognizing a specific CHC profile. An alternative explanation may be preimaginal learning of colony odor [74,75] and consequently a preformed template before adult emergence. Upon emergence the callow ants are groomed by their nestmate nurse-ants, thereby experiencing the first acquisition of nestmate CHC composition. It is assumed that template formation is then initiated. A few days post-emergence the ant starts both producing its own CHC and exchanging them with nestmates, acquiring the colony odor and concomitantly the “colony template”. The concomitant formation of the signal and the template suggests that the input and processing are likely to share the same neural routes from the peripheral olfactory organs to the central nervous system: first to the olfactory primary center, the ALs, and subsequently to the learning and memory center, the MB.

New ideas on how signature mixtures are processed by taking account of generalization of similar structures of odorant molecules have been proposed recently [76,77], but we are still far from understanding the neural mechanism underlying the comparative processing between the neural representations of the template and the encountered CHC odor information. Neural representation of the odor information provided by various CHC mixtures of nestmates and non-nestmates was studied using calcium imaging at the ALs of workers of *C.* *floridanus* [67,78]. The paired ALs of this species constitute the site where the axonal terminals of the ORNs expressing the identical ORNs derived from olfactory sensilla throughout the antennae converge, respectively. In each glomerulus, these ORNs transfer qualitatively identical neuronal signs to the projection neurons and interact with the local interneurons via synapsis. The imaging studies comprised comparative analyses of spatial patterns of neural activity in the glomeruli, when the antennae were stimulated with warmed vapors of nestmate or non-nestmate CHCs. This elicited spatial activity patterns distributed across different AL compartments, while the activity patterns in response to nestmate and non-nestmate colony odors were overlapping, as expected, because the CHC profiles of both are only qualitatively different. Moreover, the measured activity pattern variability was higher in response to repeated nestmate colony odor stimulation than to that from repeated non-nestmates, which may indicate neuronal plasticity within the olfactory system, a necessity for template reformation. It was further concluded that information regarding colony odors is processed in parallel by different neuroanatomical compartments, using the computational power of the entire AL network. Parallel processing might be advantageous, allowing reliable discrimination of highly complex social odors In *C. japonicus* the situation seems to be different. The projection neurons from the T6 and non-T6 regions terminate at different localities in the calyses of the MB and the LH, indicating that the role of the T6 glomerulus is different, presumably involved in social behavior like nestmate recognition [69].

## 5. The Pre-Filter Hypothesis for Explaining Nestmate Recognition

The prefilter hypothesis suggests that the CHC specific receptor neurons when chronically exposed to a specific CHC blend (that of a nestmate) undergo sensory adaptation and do not fire. Upon exposure of non-nestmate CHC adaptation is alleviated and firing occurs. It is suggested that this adaptation provides the first discriminatory step in nestmate recognition.

Upon encounter, ants exhibit prolonged antennation enabling access to the non- or barely-volatile CHCs. The uniqueness of the CHC sensillum lays in its action as an “olfactory contact sensillum”, due to its ability to perceive these long-chain hydrocarbons, which function as stimulus odorants for nestmate recognition in ants [24,25,26,27,28,29,79]. Electrophysiological response from a single CHC sensillum was successfully recorded using the tip-recording method that has been long been used for impulse recording from a single gustatory sensillum in many insect species [80]. Since the ant CHCs are water-insoluble, they were made soluble using a water-based solution containing a carrier protein, CjapCSP7 or Triton X-100 as mild detergent, and then contacted with the target sensillar tip, using a glass capillary recording electrode. The recorded impulse-arrays consisted of multiple, differently shaped impulse-units that originated from the different ORNs. If the receptor neurons within a sensillum, which assumes different functional roles depending on their unique receptor molecules, have adequate electrophysiological features, respectively, they are generally expected to exhibit their own shapes of impulse units, which draw distinguishable time courses of action potentials with each other. (It should be noted that even with the most sophisticated software [81], only up to 20 impulse units of different shapes can be distinguished from each other [81]).

Theoretically, the informational content of a recognition pheromone is translated to on and off combinations of impulse units that are generated by the total number of ORNs in a CHC sensillum, if those ORNs express functionally different CHC receptor genes, respectively, which in turn corresponds to the odotopes that are present in the perceived CHC blends. Indeed, electron micrographs indicated that each CHC sensillum houses 100–200 ORNs [27], enabling discrimination between 2^100–200^ qualitatively different CHC blends including unfamiliar (whether con- or heterospecific) ones, thus providing the sensilla with the potential for functioning as a nestmate recognition perception system [26]. As described above, the ORNs in the olfactory sensillum express specific olfactory receptor proteins, which correspondingly bind characteristic spectra of odotopes, *i.e.*, submolecular structural features of odorants, in accordance with general principles of olfactory perception [82,83]. Putative odotopes present in ant CHCs constitute various lengths of carbon chains, unsaturated bonds including their geometric isomer and positions, methyl branches and their positions, chirality *etc.* Perception of the different odorants in a blend by the CHC sensillum can be complex. For example, a single odorant molecule that possesses multiple odotopes may stimulate multiple ORNs, while on the other hand several different odorants that share a common odotope, can stimulate the same ORN. This is in line with the general model of the combinatory molecules that may be deciphered by the odor receptors as scent described for both vertebrates and invertebrates [84,85,86]. Indeed, the ORNs in a CHC sensillum generate particular impulse units, in line with the above assumption. The magnitude of response of an ORN is represented by the firing frequency of such an ORN-specific impulse unit, which in turn depends on the receptor membrane potential evoked by the total effects of the stimulatory odotopes approaching the ORN-specific receptor protein. Thus, the recording of the impulse arrays generated by a particular single CHC sensillum includes multiple impulse units. Therefore, the firing frequencies of the ORN-specific impulse units derived from stimulated ORNs represent quantitative information regarding the stimulatory odotopes in the CHC blends, respectively.

**Figure 1:**
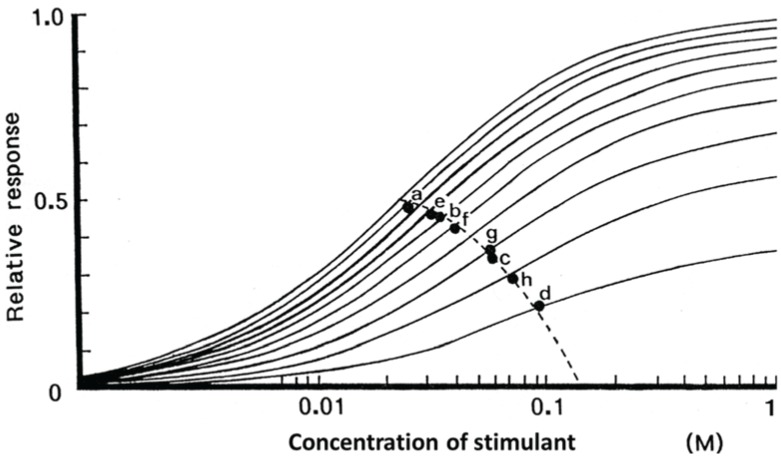
Theoretical concentration-response curves in an adapted single chemoreceptor cell, calculated according to Beidler [91] equation of R/R_m_ = 1/(1 + C_1/2_/[S]), where R/R_m_ is relative response to the maximum response; C_1/2_ is mid-point concentration; [S] is the concentration of the stimulant. The concentration-response curves are drawn at various adapted states. As the ratio of a receptor activation/inactivation-dependent receptive membrane conductance to leak conductance, (rg/G, where r, number of functional receptor molecule; g, conductance change per activated receptor molecule; G, leak conductance at the receptive membrane) is changed to be 100, 90, 80, 70, 60, 50, 40, 30, 20, and 10% of that at the non-adapted state by sensory adaptation, C_1/2_ increases and responsiveness of the cell is reduced but the sensory resolution increases, especially for high concentration range. A dotted line follows mid-points of the theoretical concentration-response curves. Experimentally obtained mid-points in a variously adapted sugar taste receptor cell of the blowfly (a–h) are well fitted to the dotted line (Ozaki, unpublished work [92]).

The CHC sensilla of *C. japonicus*, when stimulated with a non-nestmate CHC blend, but not with a nestmate CHC blend, respond with complex firing patterns comprising several impulse units derived from multiple ORNs. Accordingly, we hypothesize that the CHC sensilla of *C. japonicus* workers act as a prefilter, through which the signal emanating from a nestmate worker is cut off at the peripheral level of this sensory system, whereas that emanating from a non-nestmate worker is passed on to the brain. In considering a putative mechanism for explaining the lack of response to the colony-specific nestmate CHC blend at least in *C. japonicus*, two facts should be taken into account: (1) There is high congruency between nestmate and self-CHC blends [27]; and (2) self-CHC continuously penetrate into the sensillar cavity, being preserved in the sensillar lymph at the original ratio in a CSP-bound form [27], and therefore continuously stimulate the CHC sensillar ORNs. Thus, we hypothesized that the CHC sensillar ORNs of worker ants undergo desensitization, or sensory adaptation through the continuous exposure to the self-CHC blend. The magnitude of the desensitization evidently depends on the relative amounts of the stimulant odotopes comprising the self-CHC blend (Figure 1). In the case of a nestmate blend there is good congruency both in the numbers and relative amounts of odotopes with those of the self-CHC blend. Blends of non-nestmates, on the other hand, differ at least in the relative proportions of these odotopes, thus eliciting a response, *i.e.*, firing and transmitting the information to the brain. It is assumed that the amounts of CHC on the cuticle, at least within the same species, is near to equal or at least with very low variation, enabling an accurate assessment of the variation in the proportion of the various blend components [87]. Sensory adaptation, however, may not be the only mechanism. Results of a study with *Camponotus floridanus* where the temporal response of the ants that were habituated to a non-nestmate hydrocarbon blend fits with neural template reformation rather than sensory adaptation [88]. Further negating the sensory adaptation hypothesis was the findings that stimulating the ants with vaporized (by warming) nestmate CHCs elicited activation of antennal lobes [89]. It is possible that in *C. floridanus* the prefilter is not as efficient as in *C. jponicus*, and may be leaky similar to what was found for the supercolony-forming ant *Formica yessensis* [26]. Otherwise, it is possible that ants have two types of CHC sensilla; one is highly tuned to CHCs and the other is not, as was proposed by [38], which reconciles the discrepancy between the two studies.

The hypothetical phenomenon in a partially adapted ORN can be compared to light adaptation in a photoreceptor cell to constant background light [90]. The log intensity-response curve of a photoreceptor cell when adapting to a continuous background light indicates a decrease in the absolute sensitivity, but an increase in the sensory resolution, especially for additional intensity range. In analogy, we hypothesize that the CHC sensillar ORN that is desensitized by self-CHC blend-odotopes becomes non-responsive, both qualitatively and quantitatively, to nestmate blends, but will at the same time increase its sensory resolution for even a slight increase in any of the odotope quantities that occurs in a non-nestmate CHC blend (Figure 1). This hypothetical system, based on continuous adaptation to the self-CHC odor should enable the ants to discriminate non-nestmates from nestmates even on the basis of a small difference in CHC blends. An exception is when the opponent possesses lower amounts of each and every odotope in its CHC blend compared to the self-CHC blend, which is highly unlikely to occur.

We do not know what the molecular basis of sensory adaptation is in ants, but we assume it to be one of those described for other sensory systems: reversible receptor inactivation via phosphorylation by Rhodopsin kinase in the visual system [93,94]; by protein kinase C in the gustatory system [95]; and modulation of cAMP-gated channel or adenylate cyclase by Ca^2+^ in the olfactory system [96,97]. Regarding odor perception, it is worth reiterating that in *C. japonicus* the chemosensory protein CSP7 was able to solubilize CHC in a buffer solution is a blend-specific manner. This enabled the presentation of the exact blend penetrating the basiconic sensilla to the ORNs.

Upon perception, the informational content of the opponent’s CHC is qualitatively translated into a combination of impulse-units generated by the appropriate ORNs, as well as quantitatively through the frequency of every impulse unit, both of which are then transferred for further neural processes. Thus, a perfect prefilter system should block the information transfer of nestmate CHC blend to the AL, the primary olfactory center, and subsequently to the higher brain but not that of non-nestmates. Corroborating this hypothesis are electrophysiological recordings for at least two ant species indicating that stimulating the antennae with non-nestmate CHCs elicit firing of the ORNs, whereas when stimulated with nestmate CHCs the ORNs remain almost invariably silent [26,27]. In honeybees, it is known that odor concentration affects odor identity [98]. This raises the idea that responsiveness of the olfactory system for nestmate and non-nestmate discrimination in ants may be not only qualitatively- but also quantitatively-dependent on the CHC blend. In our investigation of *C. japonicus*, regardless of nestmate or non-nestmate CHC blend, both the magnitude of aggressive behavior and the number of workers showing aggressive behavior increased in a dose-dependent manner for a particular range of CHC amounts. However, mean value of the threshold CHC amount, which was defined as the CHC amount inducing aggressive behavior in 50% of test workers, was about one log unit higher for nestmate blend than for non-nestmate blend [99]. On the other hand, such a difference in the threshold CHC amount between supercolonymate and non-supercolonymate blends was a little less than one log unit [26]. Thus, it is hypothesized that if differences in threshold CHC amount between nestmate and non-nestmate blends are large, the discrimination ability should accordingly be high and vice versa. This evidently also depends on species. Moreover, it was suggested that individual variability of aggressiveness is not negligible and that experimental regulation of test CHC amount is important.

## 6. Behavior-Switching Threshold Hypothesis as Complementing the Sensory Prefilter Hypothesis

In some species, like *C. japonicus*, the prefilter functions very well at the sensory level, resulting in a clear-cut distinction of nestmate *versus* non-nestmate by the perceiving ant. However, as evident in the study of *Formica yessensis*, this pre-filtration might be incomplete. Workers of this supercolony-forming species exhibit strong aggression towards workers of other species (e.g., *C. japonicus*), but only moderately aggressive behavior towards conspecific heterocolonial workers (less than 50% of workers exhibit aggressive behavior), even lesser aggression (up to 20%) toward non-nestmates within their own supercolony (colony-mates), and no aggression at all among nestmates. However, a certain portion of the CHC sensilla (25%–30% of tested sensilla) responds even to nestmate CHC blend. Thus, the impulses elicited by the nestmate CHC blend reach the brain, but do not elicit any aggressive behavior. In order to explain this seeming discrepancy between neural activity and aggressive behavior, Kidokoro-Kobayashi *et al.* [26] hypothesized the existence of an aggressive-behavior-switching center in the brain that is switched on only by above-threshold neural activity. They further postulated that the low intensity of neural activity generated by stimulation with nestmate CHC is not sufficient to elicit aggressive behavior. In the case of *F. yessensis*, the behavioral data suggest that the behavior-switching center triggers aggressive behavior in an all or none manner. However, in other ant species the response may be modulated by experience or in a context-dependent manner, which may reset the threshold [95,96]. As described above, neuroanatomical studies with *C. japonicus* have suggested that the information from the CHC sensillar ORNs that converge to the T6 glomeruli (presumably conveying information pertaining to social phenomena) is sent on to the LH-II by projection neurons along the m-ACT, whereas information that converges to the non-T6 glomeruli (presumably conveying more general information) is sent on to the LH-I [69]. Subsequently, the information processed in the LH can be transferred to the descending neurons innervating the thoracic ganglion, which, in turn, could directly, or indirectly via interneurons, connect to motor neurons that may be involved in aggressive behavior, such as rushing leg movement or abdomen curling. Recently, odor- and pheromone-specific neural activity patterns have been successfully recorded from the honeybee LH [100], rendering it a good candidate for the hypothetical aggressive-behavior-switching center.

Another possible brain area for the aggressive-behavior switching center is that of the MB. In insects, the MB are important for olfactory learning and memory [101]. It was shown in *Drosophila melanogaster*, by transiently blocking synaptic transmission during the different phases of memory processing, that MB signaling is required during memory retrieval but not during acquisition or consolidation [102]. This suggests that MB signaling may be required for template storage or reforming but is less necessary for template acquisition or recognition signal/template matching. Moreover, a central element in olfactory decoding in insects is that of feedback signals from the MB to the AL [103]. We suggest that such a feedback loop may be involved in the plasticity and resettability of the aggressive-switching center. A neuronal pathway can be postulated. The T6 region can be considered to be a cross-road, where information passing through the prefilter, whether completely or partially, accumulates and is sent concomitantly to both the LH-II and to a special part of the MB calyx called the lp-I via m-APT.

The MB may also play a role in the context-dependent aggressive reaction often observed in ants. The information that is routed via the T6 glomeruli to the MB can be memorized as being associated with aggressive encounter. Such associative learning can probably be achieved, for example, through the perception of the CHC blends of non-nestmate neighbors, with which there is a high probability of encounter. It further enables the ant to respond to the CHC sensing in a context dependent manner, explaining why worker ants exhibit greater aggressive behavior toward familiar non-nestmates than unfamiliar non-nestmates (nasty neighbor effect) as reported in *Pristomirmex pungens* [104] and *Cataglyphis fortis* [105]. A resettable threshold can also accommodate the phenomenon of “dear enemy effect” [106,107]. In this case familiarity with the neighbors’ CHC through repeated encounters can result in raising the aggression-eliciting threshold, and although the different CHC blends can be perceived by the peripheral sensory system and sent to the central nervous system, the high threshold of the aggression-eliciting center will induce only a mild or no response. Thus, plasticity and resettability of the aggression-eliciting system seems to be highly adaptive.

Noteworthy is the finding that the threshold amount of a CHC blend for eliciting aggressive behavior may differ individually, as demonstrated in *F. yessensis* [26]. While it is possible that differences in the efficacy of the prefilter lie at the basis of such individuality, it is also possible that the behavior-switching threshold is subject to individual variation.

## 7. Concluding Remarks

Nestmate recognition is a pivotal phenomenon in social insects and the primary factor affecting colony cohesion and colony integration. In the social insects it is mediated by chemical signals that are colony specific. Since nestmate recognition is highly adaptive in the interactions among conspecifics, it imposes an evolutionary constraint on the nature of the signal, relying on quantitative (proportion of the signal components), rather than on qualitative variation (the occurrence of new compounds in the blend) in the signal. This dictates that the signal should be highly complex. Indeed, the CHC, the putative recognition signal in ants, comprises a very complex blend. Signal complexity has undoubtedly necessitated the evolution of an appropriately complex perception and deciphering neural system: a multiple receptor system to accommodate the multiple components in the blend and a sophisticated neural network to convey the information to the brain.

In terms of perception, the parsimonious solution for sensory integration is to possess a single sensillum that houses multiple receptors, to match the multiple compounds composing the signal. Evidence for such a solution emerged in the identification of the specific basiconic CHC sensillum [27]. Admittedly, this is only one case, and corroboration of this hypothesis must await further studies in additional social insect species.

In terms of deciphering the information, there are two hypotheses, which although seemingly contradictory may not be mutually exclusive and can occur in a species-specific manner. The first, classical hypothesis, is that of a neural template, at the core of which is that *all* the chemical information that reaches the peripheral sensory system (the antennae) is passed to the AL, where matching occurs. The alternative hypothesis posits that the peripheral system acts as a prefilter, transmitting only non-nestmate signals while blocking those of nestmates.

Evidence for a neural template has been provided from calcium imaging of the AL after stimulating the antennae with nestmate or non-nestmate CHC (made volatile by heating) [78,89]; but the results were equivocal. Another line of evidence comes from the dynamics in changes in behavior (nestmate recognition assay) after painting the ant’s antennae with either a nestmate’s or non-nestmate’s PPG secretion [88]. There was no change in the ant’s behavior within 2 hours post-treatment, whereas after 15 hours the treated ant’s behavior had changed as predicted: showing no aggression either towards its natural nestmates or towards nestmates of the ants from which the PPG secretion had been taken (originally non-nestmate). The authors concluded that such dynamics exclude the possibility of sensory adaptation at the antennal level and are in line with a neural template reformation.

Evidence for the prefilter hypothesis comes from the results of single sensillar recording from the CHC sensillum. While firing when stimulated with non-nestmate CHC (delivered directly onto the target sensillum as a CHC solution made soluble in buffer using a CSP carrier protein or Triton × emulsifier), it remained silent when stimulated with nestmate CHC solution. To ensure that the phenomenon observed was not the result of some feedback arising from the brain (e.g., the AL) this experiment was repeated with excised antennae, and gave the same results. The involvement of the brain, according to the prefilter hypothesis, is thus not by a matching/mismatching between signal and template but by comprising a threshold-dependent behavior-switching center, the location of which is postulated to be either in the LH or the MB. The AL accordingly serves to accumulate and amalgamate the sensory output from the antennae, rather than having a discriminatory function.

In conclusion, the process of deciphering nestmate signaling seems to be complex one and presents a challenge to neurobiologists as well as social insect researchers.

## References

[1] Blum M.S. (1996). Semiochemical parsimony in the Arthropoda. Annu. Rev. Entomol..

[2] Bradshaw J., Howse P., Bell W., Carde R. (1984). Sociochemicals of ants. Chemical Ecology of Insects.

[3] Morgan E.D., Lewis T. (1984). Chemical words and phrases in the language of pheromone for foraging and recruitmen. Insect Communication.

[4] Hölldobler B., Wilson E. (1990). The Ants.

[5] Karlson P., Luscher M. (1959). “Pheromones”: A new term for a class of biologically active substances. Nature.

[6] Wyatt T.D. (2010). Pheromones and signature mixtures: Defining species-wide signals and variable cues for identity in both invertebrates and vertebrates. J. Comp. Physiol. A Neuroethol. Sens. Neural Behav. Physiol..

[7] Bradshaw J.W., Baker R., Howse P.E. (1979). Multicomponent alarm pheromone in the mandibular gland of major workers of the African weaver ant *Oecophyla longinoda.*. Physiol. Entomol..

[8] Hefetz A., Graur D. (1988). The significance of multicomponent pheromones in denoting specific compositions. Biochem. Syst. Ecol..

[9] Lenoir A., Fresneau D., Errard C., Hefetz A., Detrain C., Deneubourg J.L., Pasteels J.M. (1999). Individuality and colonial identity in ants: The emergence of the social representation concept. Information Processing in Social Insects.

[10] Hefetz A. (2007). The evolution of hydrocarbon pheromone parsimony in ants (Hymenoptera: Formicidae)—Interplay of colony odor uniformity and odor idiosyncrasy. A review. Myrmecol. News.

[11] Van Zweden J.S., d’Ettorre P., Blomquist G.J., Bagnères A.-G. (2010). Nestmate recognition in social insects and the role of hydrocarbons. Insect Hydrocarbons: Biology, Biochemistry, and Chemical Ecology.

[12] Vander Meer R.K., Morel L., Vander Meer R.K., Breed M., Winston M., Espelie K.E. (1998). Nestmate recognition in ants. Pheromone Communication in Social Insects: Ants, Wasps, Bees and Termites.

[13] Brandstaetter A.S., Endler A., Kleineidam C.J. (2008). Nestmate recognition in ants is possible without tactile interaction. Naturwissenschaften.

[14] Hadley N.F. (1994). Water Relations of Terrestrial Arthropods.

[15] Rourke B.C., Gibbs A.G. (1999). Effects of lipid phase transitions on cuticular permeability: Model membrane and *in situ* studies. J. Exp. Biol..

[16] Gibbs A.G., Markow T.A. (2001). Effects of age on water balance in *Drosophila* species. Physiol. Biochem. Zool..

[17] Wagner D., Tissot M., Gordon D. (2001). Task-Related environment alters the cuticular hydrocarbon composition of harvester ants. J. Chem. Ecol..

[18] Le Conte Y., Hefetz A. (2008). Primer pheromones in social hymenoptera. Annu. Rev. Entomol..

[19] Peeters C., Liebig J., Gadau J., Fewell J. (2009). Fertility signaling as a general mechanism of regulating reproductive division of labor in ants. Organization of Insect Societies: From Genome to Sociocomplexity.

[20] Greene M.J., Gordon D.M. (2007). Structural complexity of chemical recognition cues affects the perception of group membership in the ants *Linephithema humile* and *Aphaenogaster cockerelli*. J. Exp. Biol..

[21] Greene M.J., Gordon D.M. (2003). Cuticular hydrocarbons inform task decisions. Nature.

[22] Peeters C., Monnin T., Malosse C. (1999). Cuticular hydrocarbons correlated with reproductive status in a queenless ant. Proc. R. Soc. B Biol. Sci..

[23] Van Oystaeyen A., Oliveira R.C., Holman L., van Zweden J.S., Romero C., Oi C.A., d’Ettorre P., Khalesi M., Billen J., Wäckers F. (2014). Conserved class of queen pheromones stops social insect workers from reproducing. Science.

[24] Lahav S., Soroker V., Hefetz A., Vander Meer R.K. (1999). Direct behavioral evidence for hydrocarbons as ant recognition discriminators. Naturwissenschaften.

[25] Wagner D., Tissot M., Cuevas W., Gordon D.M. (2000). Harvester ants utilize cuticular hydrocarbons in nestmate recognition. J. Chem. Ecol..

[26] Kidokoro-Kobayashi M., Iwakura M., Fujiwara-Tsujii N., Fujiwara S., Sakura M., Sakamoto H., Higashi S., Hefetz A., Ozaki M. (2012). Chemical discrimination and aggressiveness via cuticular Hydrocarbons in a supercolony-forming ant, *Formica yessensis*. PLoS One.

[27] Ozaki M., Wada-Katsumata A., Fujikawa K., Iwasaki M., Yokohari F., Satoji Y., Nisimura T., Yamaoka R. (2005). Ant nestmate and non-nestmate discrimination by a chemosensory sensillum. Science.

[28] Akino T., Yamamura K., Wakamura S., Yamaoka R. (2004). Direct behavioral evidence for hydrocarbons as nestmate recognition cues in *Formica japonica* (Hymenoptera:Formicidae). Appl. Entomol. Zool..

[29] Brandt M., van Wilgenburg E., Sulc R., Shea K.J., Tsutsui N.D. (2009). The scent of supercolonies: The discovery, synthesis and behavioural verification of ant colony recognition cues. BMC Biol..

[30] Crozier R.H., Pamilo P. (1996). Evolution of Social Insect Colonies: Sex allocation and kin selection.

[31] Sherman P.W. (1985). Alarm calls of Belding’s ground squirrels to aerial predators: Nepotism or self-preservation?. Behav. Ecol. Sociobiol..

[32] Smith S.M. (1975). Innate recognition of coral snake pattern by a possible avian predator. Science.

[33] Rich T.J., Hurst J.L. (1999). The competing countermarks hypothesis: Reliable assessment of competitive ability by potential mates. Anim. Behav..

[34] Rich T.J., Hurst J.L. (1998). Scent marks as reliable signals of the competitive ability of mates. Anim. Behav..

[35] Zala S.M., Potts W.K., Penn D.J. (2004). Scent-Marking displays provide honest signals of health and infection. Behav. Ecol..

[36] Sherman P.W., Reeve H.K., Pfennig D., Krebs J.R., Davies N.B. (1999). Recognition systems. Behavioral Ecology.

[37] Breed M.D., Bennett B., Fletcher D.J.C., Michener C.D. (1987). Kin recognition in highly eusocial insects. Kin Recognition in Animals.

[38] Bos N., d’Ettorre P. (2012). Recognition of social identity in ants. Front. Psychol..

[39] Crozier R.H., Fletcher D.J.C., Michener C.D. (1987). Genetic aspects of kin recognition: models for innate components of colony odor. Social Hymenoptera.

[40] Crozier R.H., Dix M.W. (1979). Analysis of two genetic models for the innate components of colony odour in social Hymenoptera. Behav. Ecol. Sociobiol..

[41] Hamilton W.D. (1964). The genetical evolution of social behaviour. I, II. J. Theor. Biol..

[42] Hamilton W.D. (1972). Altruism and related phenomena mainly in the social insects. Annu. Rev. Ecol. Syst..

[43] Fletcher D.J.C., Michener C.D.E. (1987). Kin Recognition in Animals.

[44] Hughes W.O.H., Oldroyd B.P., Beekman M., Ratnieks F.L.W. (2008). Ancestral monogamy shows kin selection is key to the evolution of eusociality. Science.

[45] Soroker V., Vienne C., Hefetz A., Nowbahari E. (1994). The postpharyngeal gland as a “gestalt” organ for nestmate recognition in the ant *Cataglyphis niger*. Naturwissenschaften.

[46] Le Moli F., Mori A. (1985). The influence of the early experience of worker ants on enslavement. Anim. Behav..

[47] Errard C. (1994). Development of interspecific recognition behavior in the ants *Manica rubida* and *Formica selysi* (Hymenoptera: Formicidae) reared in mixed-species groups. J. Insect Behav..

[48] Errard C., Hefetz A. (1997). Label familiarity and discriminatory ability of ants reared in mixed groups. Insectes Soc..

[49] Errard C. (1986). Role of early experience in mixed-colony odor recognition in the ants *Manica rubida* and *Formica selysi*. Ethology.

[50] Hefetz A., Errard C., Cojocaru M. (1992). Heterospecific substances in the postpharyngeal gland of ants reared in mixed groups. Naturwissenschaften.

[51] Errard C., Hefetz A., Jaisson P. (2006). Social discrimination tuning in ants: Template formation and chemical similarity. Behav. Ecol. Sociobiol..

[52] Vander Meer R., Saliwanchik D., Lavine B. (1989). Temporal changes in colony cuticular hydrocarbon patterns of *Solenopsis invicta*. Implications for nestmate recognition. J. Chem. Ecol..

[53] Lahav S., Soroker V., Vander Meer R.K., Hefetz A. (2001). Segregation of colony odor in the desert ant *Cataglyphis niger*. J. Chem. Ecol..

[54] Errard C. (1994). Long-Term memory involved in nestmate recognition in ants. Anim. Behav..

[55] Hojo M.K., Yamamoto A., Akino T., Tsuji K., Yamaoka R. (2014). Ants use partner specific odors to learn to recognize a mutualistic partner. PLoS One.

[56] Bos N., Guerrieri F.J., d’Ettorre P. (2010). Significance of chemical recognition cues is context dependent in ants. Anim. Behav..

[57] Soroker V., Hefetz A., Cojocaru M., Billen J., Franke S., Francke W. (1995). Structural and chemical ontogeny of the postpharyngeal gland in the desert ant *Cataglyphis niger*. Physiol. Entomol..

[58] Steinbrecht R.A. (1999). Olfactory Receptors, Atlas of Arthropod Sensory Receptors, Dynamic Morphology in Relation to Function.

[59] Nakanishi A., Nishino H., Watanabe H., Yokohari F., Nishikawa M. (2009). Sex-Specific antennal sensory system in the ant *Camponotus japonicus*: Structure and distribution of sensilla on the flagellum. Cell Tissue Res..

[60] Pelosi P., Zhou J.J., Ban L.P., Calvello M. (2006). Soluble proteins in insect chemical communication. Cell. Mol. Life Sci..

[61] Gotzek D., Robertson H.M., Wurm Y., Shoemaker D. (2011). Odorant binding proteins of the red imported fire ant, *Solenopsis invicta*: An example of the problems facing the analysis of widely divergent proteins. PLoS One.

[62] Kulmuni J., Havukainen H. (2013). Insights into the evolution of the CSP gene family through the integration of evolutionary analysis and comparative protein modeling. PLoS One.

[63] Kulmuni J., Wurm Y., Pamilo P. (2013). Comparative genomics of chemosensory protein genes reveals rapid evolution and positive selection in ant-specific duplicates. Heredity.

[64] Ishida Y., Tsuchiya W., Fujii T., Fujimoto Z., Miyazawa M., Ishibashi J., Matsuyama S., Ishikawa Y., Yamazaki T. (2014). Niemann-Pick type C2 protein mediating chemical communication in the worker ant. Proc. Natl. Acad. Sci. USA.

[65] Chapman R.F. (1998). The Insects: Structure and Function.

[66] Schönitzer K., Lawitzky G. (1987). A phylogenetic study of the antenna cleaner in Formicidae, Mutillidae, and Tiphiidae (Insecta, Hymenoptera). Zoomorphology.

[67] Zube C., Kleineidam C.J., Kirschner S., Neef J., Rossler W. (2008). Organization of the olfactory pathway and odor processing in the antennal lobe of the ant *Camponotus floridanus*. J. Comp. Neurol..

[68] Zube C., Rössler W. (2008). Caste- and sex-specific adaptations within the olfactory pathway in the brain of the ant *Camponotus floridanus*. Arthropod Struct. Dev..

[69] Nishikawa M., Watanabe H., Yokohari F. (2012). Higher brain centers for social tasks in worker ants, *Camponotus japonicus*. J. Comp. Neurol..

[70] Laissue P.P., Reiter C., Hiesinger P.R., Halter S., Fischbach K.F., Stocker R.F. (1999). Three-Dimensional reconstruction of the antennal lobe in *Drosophila melanogaster*. J. Comp. Neurol..

[71] Galizia C.G., Menzel R. (2001). The role of glomeruli in the neural representation of odours: Results from optical recording studies. J. Insect Physiol..

[72] Stroeymeyt N., Guerrieri F.J., van Zweden J.S., d’Ettorre P. (2010). Rapid decision-making with side-specific perceptual discrimination in ants. PLoS One.

[73] Kaba H., Rosser A., Keverne B. (1989). Neural basis of olfactory memory in the context of pregnancy block. Neuroscience.

[74] Signorotti L., Jaisson P., d’Ettorre P. (2014). Larval memory affects adult nest-mate recognition in the ant *Aphaenogaster senilis*. Proc. R. Soc. B Biol. Sci..

[75] Isingrini M., Jaisson P., Lenoir A., Passera L., Lachaud J.-P. (1986). Influence of preimaginal experience on the social sdult ants and the importance fellow in nestmate recognition. The Individual and Society.

[76] Bos N. (2014). Asymmetry in olfactory generalization and the inclusion criterion in ants. Commun. Integr. Biol..

[77] Bos N., D’Ettorre P., Guerrieri F.J. (2013). Chemical structure of odorants and perceptual similarity in ants. J. Exp. Biol..

[78] Brandstaetter A.S., Kleineidam C.J. (2011). Distributed representation of social odors indicates parallel processing in the antennal lobe of ants. J. Neurophysiol..

[79] Thomas M.L., Parry L.J., Allan R.A., Elgar M.A. (1999). Geographic affinity, cuticular hydrocarbons and colony recognition in the Australian meat ant *Iridomyrmex purpureus*. Naturwissenschaften.

[80] Hodgson E.S., Lettvin J.Y., Roeder K.D. (1955). Physiology of a primary chemoreceptor unit. Science.

[81] (2011). SpikeTaro.

[82] Breer H. (2003). Olfactory receptors: Molecular basis for recognition and discrimination of odors. Anal. Bioanal. Chem..

[83] Krieger J., Breer H. (1999). Olfactory reception in invertebrates. Science.

[84] Buck L.B. (1995). Unraveling chemosensory diversity. Cell.

[85] Sachse S., Rappert A., Galizia C.G. (1999). The spatial representation of chemical structures in the antennal lobe of honeybees: Steps towards the olfactory code. Eur. J. Neurosci..

[86] Wang J.W., Wong A.M., Flores J., Vosshall L.B., Axel R. (2003). Two-Photon calcium imaging reveals an odor-evoked map of activity in the fly brain. Cell.

[87] Soroker V., Lucas C., Simon T., Fresneau D., Durand J.L., Hefetz A. (2003). Hydrocarbon distribution and colony odour homogenisation in *Pachycondyla Apicalis*. Insectes Soc..

[88] Leonhardt S.D., Brandstaetter A.S., Kleineidam C.J. (2007). Reformation process of the neuronal template for nestmate-recognition cues in the carpenter ant *Camponotus floridanus*. J. Comp. Physiol. A.

[89] Brandstaetter A.S., Rossler W., Kleineidam C.J. (2010). Dummies *versus* air puffs: Efficient stimulus delivery for low-volatile odors. Chem. Senses.

[90] Dawis S.M., Purple R.L. (1982). Adaptation in cones: A general model. Biophys. J..

[91] Beidler L.M. (1954). A theory of taste stimulation. J. Gen. Physiol..

[92] Ozaki M. (1999).

[93] Maeda T., Imanishi Y., Palczewski K. (2003). Rhodopsin phosphorylation: 30 Years later. Prog. Retin. Eye Res..

[94] Kohout T.A., Lefkowitz R.J. (2003). Regulation of G protein-coupled receptor kinases and arrestins during receptor desensitization. Mol. Pharmacol..

[95] Ozaki M., Amakawa T. (1992). Adaptation-promoting effect of IP3, Ca^2+^, and phorbol ester on the sugar taste receptor cell of the blowfly, *Phormia regina*. J. Gen. Physiol..

[96] Kurahashi T., Menini A. (1997). Mechanism of odorant adaptation in the olfactory receptor cell. Nature.

[97] Matthews H.R., Reisert J. (2003). Calcium, the two-faced messenger of olfactory transduction and adaptation. Curr. Opin. Neurobiol..

[98] Boulay R., Katzav-Gozansky T., vander Meer R.K., Hefetz A. (2003). Colony insularity through queen control on worker social motivation in ants. Proc. R. Soc. B Biol. Sci..

[99] Roeder T. (2005). Tyramine and octopamine: Ruling behavior and metabolism. Annu. Rev. Entomol..

[100] Roussel E., Carcaud J., Combe M., Giurfa M., Sandoz J.-C. (2014). Olfactory coding in the honeybee lateral horn. Curr. Biol..

[101] De Belle J.S., Heisenberg M. (1994). Associative odor learning in *Drosophila* abolished by chemical ablation of mushroom bodies. Science.

[102] McGuire S.E., Le P.T., Davis R.L. (2001). The role of *Drosophila* mushroom body signaling in olfactory memory. Science.

[103] Getz W.M., Lutz A. (1999). A neural network model of general olfactory coding in the insect antennal lobe. Chem. Senses.

[104] Sanada-Morimura S., Minai M., Yokoyama M., Hirota T., Satoh T., Obara Y. (2003). Encounter-induced hostility to neighbors in the ant *Pristomyrmex Pungens*. Behav. Ecol..

[105] Knaden M., Wehner R. (2003). Nest defense and conspecific enemy recognition in the desert ant *Cataglyphis Fortis*. J. Insect Behav..

[106] Heinze J., Foitzik S., Hippert A., Hoelldobler B. (1996). Apparent dear-enemy phenomenon and environment-based recognition cues in the ant *Leptothorax nylanderi*. Ethology.

[107] Dimarco R.D., Farji-Brener A.G., Premoli A.C. (2010). Dear enemy phenomenon in the leaf-cutting ant *Acromyrmex lobicornis*: Behavioral and genetic evidence. Behav. Ecol..

